# The use of mesenchymal stem cells adhered to the suture filament in the closure of rat aponeurosis

**DOI:** 10.1590/0102-67202026000002e1931

**Published:** 2026-04-20

**Authors:** Jefferson KALIL, Joaquim Murray BUSTORFF-SILVA, Davi Reis CALDERONI, Krissia Caroline LEME, Gabriela Cardoso de Arruda CAMARGO, Wagner José FÁVARO, Ângela Cristina Malheiros LUZO

**Affiliations:** 1 Universidade Estadual de Campinas, Faculty of Medical Sciences – Campinas (SP), Brazil.; 2 Universidade Estadual de Campinas, Faculty of Medical Sciences, Department of Surgery – Campinas (SP), Brazil;; 3 Universidade Estadual de Campinas, Biology Institute, Department of Structural and Functional Biology – Campinas (SP), Brazil.; 4 Universidade Estadual de Campinas, Biology Institute, Department of Structural and Functional Biology – Campinas (SP), Brazil.; 5 Universidade Estadual de Campinas, Biology Institute, Laboratory of Nerve Regeneration – Campinas (SP), Brazil.

**Keywords:** Incisional hernia, Aponeurosis, Mesenchymal stem cells, Tensile strength, Collagen, Hérnia Incisional, Aponeurose, Célula-Tronco Mesenquimais, Resistência à Tração, Colágeno

## Abstract

**Background:**

Abdominal wall hernia is a common disease, with an incidence of around 20%. Recent studies have shown the benefits of using stem cells, especially mesenchymal ones, to improve tissue healing.

**Aims:**

Evaluate the use of mesenchymal stem cells derived from adipocytes adhered to a suture filament to enhance tensile strength and collagen formation in aponeurosis.

**Methods:**

Human stem cells derived from adipocytes were adhered to a suture filament. Thirty-seven rats of the species Sprague Dawley were divided into three groups: Group 1 was the control group, Group 2 used only a regular suture filament to close abdominal aponeurosis, and Group 3 used a suture filament with stem cells. These animals were evaluated seven, 14, and 56 days after the intervention.

**Results:**

Rupture occurred at the semilunar line and the midline. All animals from Groups 2 and 3, submitted to incision and closure, evaluated at D7 and D14, showed a rupture in the midline. However, all animals evaluated at D56 (all groups) ruptured at the semilunar line. Furthermore, tensile strength was significantly lower at D7 in Groups 2 and 3 compared to Group 1 (p<0,001). On D14, Groups 2 and 3 showed a similar increase in tensile strength, but still inferior to the one observed in Group 1 (p<0,05). On D56, all groups reached similar values (p=0,074, p>0.05). Collagen histologic analysis showed that animals from Group 3 had the highest values in all time points, and Group 2 had higher values than Group 1 in all time points (p>0,05). In graphical analysis, Groups 2 and 3, on D7, had an increase in collagen, but on D14 showed a decrease, with a similar level on D56 (p>0,05).

**Conclusions:**

This study do not support the use of mesenchymal stem cells to improve the healing of a midline abdominal incision in healthy subjects. However, an option for future studies is to employ this filament, combined with matrices for reconstructive purposes, in areas requiring extensive repair, such as large hernias where the aponeurosis is insufficient for defect correction.

## ARTICLE HIGHLIGHTS

Abdominal wall hernia is a globally prevalent disease, with its subtype, incisional hernia, occurring in 10 to 20% of patients after any surgical incision involving the abdominal wall.

Stem cells have been studied for cellular therapy, as drug targets and as sources for generating different tissues for drug testing.

Specifically, adipose-derived mesenchymal stem cells have shown excellent cellular proliferation and differentiation capacity, making them excellent candidates for therapeutic use.

Use of a suture filament with adhered mesenchymal stem cells in the healing of an abdominal incision is an option in the future.

## Summary

**CENTRAL MESSAGE:**

This research aimed to use mesenchymal stem cells derived from adipocytes, attached to a suture thread, to increase tensile strength and collagen formation in the abdominal aponeurosis of rats. Human stem cells derived from adipocytes were attached to a polyglactin 910 suture thread. Thirty-seven Sprague Dawley rats were used, divided into three groups. We concluded that the use of stem cells did not provide any benefit in improving tensile strength or the amount of collagen formed at the site. Future research could be conducted to evaluate whether there would be any benefit in situations of fragility, such as malnutrition, cancer, immunosuppression, or medication use.

**PERSPECTIVES:**

The use of stem cells did not show any benefit in improving tensile strength or the amount of collagen formed at the site. However, it is possible that further studies could evaluate the use of this filament, combined with matrices for reconstructive purposes, in areas requiring extensive repairs, such as large hernias where the aponeurosis is insufficient to correct the defect. Furthermore, research could be conducted to assess whether there might be any benefit in situations of fragility, such as malnutrition, cancer, immunosuppression, or medication use.

## VISUAL ABSTRACT

**Figure:**
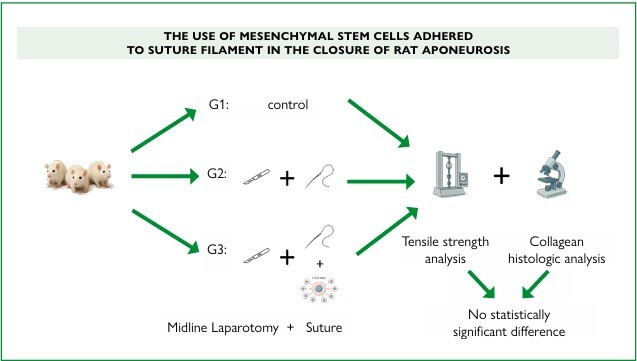

## RESUMO

**Racional:**

Hérnia da parede abdominal é uma condição comum, com incidência de aproximadamente 20%. Estudos recentes demonstraram benefícios do uso de células-tronco, especialmente as mesenquimais, para potencializar a cicatrização tecidual.

**Objetivos:**

Avaliar o uso de células-tronco mesenquimais derivadas de adipócitos, aderidas a um fio de sutura, para aumento da força tênsil e formação de colágeno na aponeurose.

**Métodos:**

Células-tronco humanas derivadas de adipócitos foram aderidas a um fio de sutura. Trinta e sete ratos da espécie *Sprague Dawley* foram divididos em três grupos: Grupo 1: controle; Grupo 2: sutura convencional no fechamento da aponeurose abdominal; Grupo 3: sutura com células-tronco. Os animais foram avaliados aos 7, 14 e 56 dias após a intervenção.

**Resultados:**

As rupturas ocorreram na linha semilunar e na linha média. Todos os animais dos Grupos 2 e 3, avaliados em D7 e D14, apresentaram ruptura na linha média; em D56, todos os grupos apresentaram ruptura na linha semilunar. A força tênsil foi significativamente menor em D7 nos Grupos 2 e 3 em comparação ao Grupo 1 (p<0,001). Em D14, houve aumento progressivo da força tênsil nos Grupos 2 e 3, mas ainda inferior ao Grupo 1 (p<0,05). Em D56, todos os grupos apresentaram valores semelhantes (p=0,074, p>0.05). Na análise histológica, o Grupo 3 apresentou maiores valores de colágeno em todos os períodos, enquanto o Grupo 2 superou o Grupo 1, embora sem significância estatística (p>0,05). A análise gráfica demonstrou aumento de colágeno em D7 nos Grupos 2 e 3, redução em D14 e estabilização em D56 (p>0,05).

**Conclusões:**

Este estudo não sustenta o uso de células-tronco mesenquimais para aprimorar a cicatrização de incisões abdominais em sujeitos saudáveis. No entanto, é possível que mais estudos avaliem o uso desse filamento, combinado com matrizes para fins reconstrutivos, em áreas que requerem reparos extensos, como grandes hérnias onde a aponeurose é insuficiente para a correção do defeito.

## INTRODUCTION

 Praxagoras of Kos, a physician from ancient Greece, was the first to consider hernia a surgical pathology, with the earliest historical reports of abdominal wall hernia dating back to the 14^th^ century BC^31^. Abdominal wall hernia is a globally prevalent disease, with its subtype, incisional hernia, occurring in 10 to 20% of patients after any surgical incision involving the abdominal wall^35^. Risk factors associated with the occurrence of incisional hernias include obesity (body mass index — BMI>25 kg/m), abdominal aortic aneurysm, and congenital disorders of connective tissue^1,14,15,18,20,38^. 

 The pathophysiology of incisional hernia is based on a multifactorial healing defect; the main factors involved are collagen deposition and the healing environment^8^. The role of collagen in wound healing, particularly in the formation of hernias, became apparent after it was shown that individuals with connective tissue disorders such as Ehlers-Danlos syndrome and Marfan syndrome exhibited a higher incidence of inguinal hernias than the general population^22,23^. Collagen is the extracellular matrix’s main protein responsible for its tensile strength^4,9,37,43^. 

 Stem cells are characterized by their ability to differentiate into various lineages and their high capacity for proliferation and self-regeneration^2^, and can also give rise to another identical and/or differentiated cell^30,36^. 

 Stem cells have been studied for cellular therapy as drug targets, and as sources for generating different tissues for drug testing. A specific type of adult stem cell, mesenchymal stem cells (MSCs), can differentiate into various lineages, exhibit immunomodulatory properties, and demonstrate regenerative potential^3,10^. The mechanisms of action of MSCs are known to involve paracrine effects through biomolecules, immunomodulatory effects, proliferative capacity, and potential differentiation into other cell lineages^5,11,17^. Preclinical studies with these cells have shown they possess an excellent capacity for tissue healing, reduce the inflammatory response, and improve many aspects of healing in various animal models^10^. 

 Specifically, adipose-derived mesenchymal stem cells have shown excellent cellular proliferation and differentiation capacity, low immunogenicity, and immunomodulatory capacity, making them excellent candidates for therapeutic use^7,19,21,28,33,44,46,47^. Clinical studies in various fields have been conducted with these cells, such as plastic surgery, orthopedics, immunology, cardiology, oral and maxillofacial surgery, dermatology, connective tissue diseases, neurology, nutrition, and metabolic diseases^16,32,34^. A recent study showed that using a suture filament with MSCs adhered to it effectively closed enterocutaneous fistulas^41^. 

 This study evaluated the role of a suture filament with adhered mesenchymal stem cells in the healing of an abdominal incision in rats. 

## METHODS

 This study was approved by the Animal Ethics Committee (number 5482-1/2020) and Human Research Ethics Committee (number 4.119.745) of the Universidade Estadual de Campinas (UNICAMP). 

 The MSCs used in the study were obtained from human adipose tissue (AT) of patients undergoing aesthetic liposuction. The lipoaspirated tissue was washed with phosphatebuffered saline (PBS), and, to isolate the MSCs from AT, enzymatic digestion was performed using collagenase type 1A (Gibco Invitrogen Corporation, Grand Island — New York — USA). After the enzymatic action of collagenase, lysis of red blood cells, and successive washes with PBS, the isolated cells were cultured in Dulbecco’s Modified Eagle Medium (DMEM) low glucose (Gibco Invitrogen) with 10% fetal bovine serum (VitroCell Embriolife, Campinas, São Paulo, Brazil). The cells were incubated at 37 °C in a humid atmosphere containing 5% CO₂. Cells expanded up to the fourth passage of culture were confirmed to be MSCs by adherence to plastic, absence of Cluster of Differentiation 34 (CD34) and CD45 expression, and positive expression of CD73, CD90, and CD105, characterized by flow cytometry using specific monoclonal antibodies. The genetic stability of these cells was assessed through karyotyping and telomerase enzyme activity (TRAPeze® Telomerase Detection Kit, S7700 — Chemicon International, USA). 

**Figure 1:**
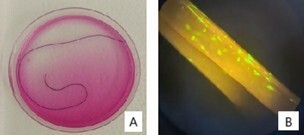
Suture Filament with Stem Cells. A: Vicryl™ filament with stem cells adhered in the culture medium. B: Live and Dead assay under fluorescent microscopy. Demonstration of living mesenchymal stem cells adhered to the suture filament.

 After the mesenchymal stem cells were prepared, they were adhered to the suture filament. The filament used was polyglactin 910, 15 cm long, with a thickness of 3-0 (Johnson and Johnson, reference J316, Vicryl™). One million MSCs were adhered to each suture filament using a glue made with thrombin and calcium gluconate in a 3:1 ratio, and they were kept in DMEM low glucose culture medium (Gibco Invitrogen) with 10% fetal bovine serum (VitroCell Embriolife, Campinas, São Paulo, Brazil). The filaments were incubated at 37 °C in a humid atmosphere containing 5% CO₂, and were observed every 24 hours. After 72 hours of culture, the filaments were used to treat the animals. To confirm the attachment of the mesenchymal stem cells to the suture filament and their viability after the fixation process with glue, a Live and Dead experiment was conducted (Invitrogen™ LIVE/DEAD™ Viability/Cytotoxicity Assay Kit — Green/Deep Red). Figure 1 shows the filament with the stem cells after preparation, immediately before animal use, and demonstrates the Live and Dead assay under fluorescent light. 

 G*Power 3.1 software, from Heinrich Heine Universität — Dussledorf, was used to calculate the sample size. Taking an elevation of 50% on tension force as clinically significant and using a significance level alpha=0.05 and a power of 0.8, it was calculated that, at each of the time points, each group should have at least four animals. As there were three time points to be studied and accounting for a 10–15% mortality of the animals, five animals were assigned to each group at each time point. As the control group would be using non-operated animals, and in the name of the 3Rs (replacement, reduction, and refinement) of animal use in experimental surgery, it was supposed that only one group of three animals would suffice to serve as a control for the whole experiment. 

 The experiment was performed with 37 male Sprague Dawley rats, aged eight weeks, weighing approximately 300 grams. All animals were housed in appropriately sized plastic boxes, with a 12-hour light/dark cycle, temperature 20±2°C, and ambient humidity, with free access to water and standard commercial food. All animals used in the study were obtained from the Multidisciplinary Center for Biological Research at UNICAMP, following approval of the study by the Animal Ethics Committee (number 54821/2020) of the UNICAMP. 

 The rats were divided into three groups:

- Group 1 (control): 3 rats;
- Group 2 (Vicryl™): 17 rats;
- Group 3 (Vicryl™ with adhered mesenchymal stem cells): 17 rats.

The day (D) of the intervention was considered D0, with evaluations conducted on days D7, D14, and D56 following the surgery (D0). 

 In Group 1, no intervention was performed; the animals were kept under the same conditions as the others. In Groups 2 and 3, the following surgical procedure was carried out: all animals (17+17) underwent general anesthesia, followed by a midline abdominal skin incision of approximately 4 cm. After the skin was opened, a 4 cm incision was made in the midline of the aponeurosis, with the peritoneum opened simultaneously, exposing the abdominal cavity. Subsequently, the aponeurosis was sutured using the following technique: a continuous suture encompassing the peritoneum and aponeurosis in a single layer, maintaining a suture-to-incision length ratio of 4:1, and using closely spaced stitches. 

 In Group 2, standard Vicryl™ 3-0 suture filament (Johnson & Johnson Medical Brazil, São Paulo, Brazil, reference J316), was used without modifications. In Group 3, the same Vicryl™ 3-0 filament, reference J316) was used, but with mesenchymal stem cells adhered to it, as described previously. The skin was then closed using Mononylon™ 4-0 filament (reference 14502, Johnson & Johnson Medical Brazil, São Paulo, Brazil), with a continuous suture in both groups. 

**Figure 2:**
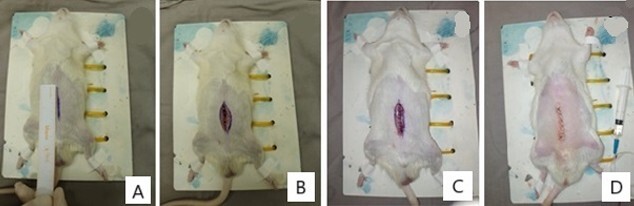
Sequence of Procedures Performed on Animals. A: Anesthetized rat; midline incision marking using a ruler; measurement of 4 cm. B: Midline incision performed with complete opening to the peritoneal cavity. C: Aponeurosis closure with "Vicryl" filament. D: Final aspect after aponeurosis and skin closure.

 Euthanasia was performed on days D7, D14, and D56 by deepening general anesthesia, using thiopental, at a dose of 25 mg intravenously, in accordance to the Animal Ethics Committee of the UNICAMP. After euthanasia, the Mononylon™ filament was removed from the skin, which was opened to expose the aponeurosis. A visual inspection detected any signs of secretion, infection, local reaction, and aponeurosis integrity. A segment of the aponeurosis, measuring 6 cm in width and 4 cm in length around the previous incision, was resected using a mold to standardize specimen collection. The segment was divided into cranial (1 cm) and caudal (3 cm) portions. The caudal segment was placed in saline solution and immediately sent for tensile strength analysis, while the cranial segment was placed in 10% buffered formalin for histological analysis. Figure 2 illustrates the procedures performed on the animals. 

 The distribution of euthanized animals was as follows:

- Group 1: three animals, with one animal at each time point (D7, D14 and D56);
- Groups 2 and 3: six animals per group at D7; six animals per group at D14; and five animals per group at D56.

The variables analyzed included the tensile strength of the aponeurosis and collagen quantification using Masson’s Trichrome and Picrosirius Red staining. 

 Tensile strength tests were performed after removing the suture filament. A universal testing machine (Texture Analyzer, Model TA500) with a 500 N load cell (Lloyd Instruments) was used, operating at a constant traction speed of 0.6 mm/s. Atraumatic Collin-type gripping clamps were attached to the specimen’s extremities, with the clamp tips positioned laterally to the semilunar line. 

 For histological analysis, the aponeuroses from all animals were collected and fixed in 10% buffered formalin for 12 hours. After fixation, the tissues were washed in 70% ethanol and dehydrated in an ascending alcohol series. The fragments were cleared with xylene for two hours and embedded in plastic polymers (Paraplast Plus, St. Louis, MO, USA). The samples were sectioned using a Slee CUT 5062 RM 2165 rotary microtome (Slee Mainz, Mainz, Germany) at 5-micrometer thickness, stained with Picrosirius Red and Masson’s Trichrome, and photographed with a Leica DM2500 photomicroscope (Leica, Munich, Germany) equipped with a DFC295 camera (Leica, Munich, Germany). 

 All samples were photographed as follows: one image at 40x magnification covering the entire midline of the aponeurosis, and three sequential images at 100x magnification, scanning the midline from edge to edge. Collagen quantification was performed by counting 80 points per 100x magnification field in the three images of the midline of each sample stained with Picrosirius Red, with an area of 15,000 pixels per frame, using the grid mode of ImageJ software. 

 Statistical analyses were conducted using the JASP software, version 0.18.3 (University of Amsterdam, Netherlands). Statistical parameters included the median, mean, standard deviation, and maximum and minimum values. Analysis of variance (ANOVA) was used to analyze these parameters at different time points for continuous variables. A significance level of 5% and a statistical power of 80% were considered for determining statistical significance. In all analyses, measurements were categorized by days (D7, D14, and D56). At each time point, the three groups were compared using ANOVA. If p<0.05, a posthoc Tukey test was performed to identify which groups presented significant differences. 

## RESULTS

### Tensile strength analysis

**Figure 3:**
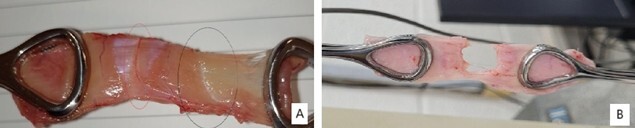
Specimen detail after rupture. A: Detail of the specimen after rupture at the semilunar line. The red circle marks the midline (no rupture), while the black circle marks the semilunar line (ruptured). B: Detail of the specimen after rupture at the midline.

 Regarding the tensile strength analysis of the aponeurosis fragments, the tissue ruptured at the semilunar line and the midline (Figure 3). 

 In Group 1, the aponeurosis of all animals ruptured at the semilunar line. In the animals subjected to intervention (Groups 2 and 3), the rupture occurred at the midline (incision site) on days D7 and D14. Finally, on D56, all animals exhibited ruptures at the semilunar line. 

**Figure 4:**
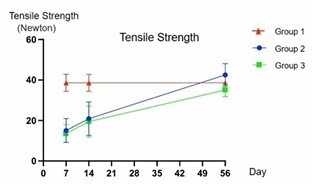
Representation of tensile strength over time: days 7, 14, and 56, comparing the groups. Tensile strength in Newtons (GraphPad Prism v10.2). D7: p<0.001; D14: p<0.05; D56: p=0.074.

 Graphical analysis of tensile strength revealed that, on D7, the tensile strength in Groups 2 and 3 was significantly lower than in Group 1 (p<0.001). A progressive and similar increase in tensile strength was observed in Groups 2 and 3 on D14. However, tensile strength remained significantly lower than in Group 1 (p<0.05). By D56, all groups exhibited very similar values, with no statistically significant differences (p=0.074). Notably, on D7 and D14, there were no significant differences between Groups 2 and 3 (p>0.05) (Figure 4). 

 Table 1 presents a descriptive analysis of tensile strength, divided by group and time point. The study compared all groups simultaneously, and, when statistical significance was detected, pairwise comparisons were performed to identify which groups exhibited significant differences. Groups 2 and 3 did not show a statistically significant difference on D7 and D14 when compared to each other (p>0.05). However, both showed a significant difference compared to Group 1 at the same time points (p<0.05). Nevertheless, on D56, there was no statistically significant difference among the groups (p>0.05) (Table 1). 

**Table 1:** Tensile strength: descriptive analysis, separated by group and period.

		Mean	Median	SD	Minimum	Maximum
Day 7	Group 1	38.63	35.56	4.22	35.56	43.44
	Group 2	15.09^&^	15.19	5.91	8.69	24.12
	Group 3	13.61^&^	14.05	4.46	7.53	17.88
Day 14	Group 1	38.63	35.56	4.22	35.56	43.44
	Group 2	20.90^&^	21.76	8.28	9.61	30.63
	Group 3	19.49^&^	18.55	7.67	10.81	29.55
Day 56	Group 1	38.63	35.56	4.22	35.56	43.44
	Group 2	42.52	41.48	5.61	37.14	51.66
	Group 3	35.06	35.24	3.23	31.69	39.48

### Histological analysis

**Figure 5:**
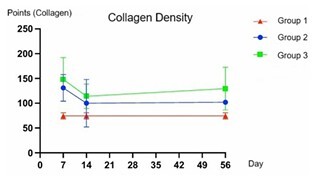
Representation of collagen density in the specimens over time: days 7, 14, and 56, comparing the groups. Points indicate the number of collagen-positive areas in the sample (GraphPad Prism v10.2). D7, D14 and D56: p>0.05.

 The graphical analysis indicated that Groups 2 and 3 experienced an increase in collagen on D7, followed by a decline on D14, and then stabilized at similar levels on D56. Despite these trends, no significant differences were observed among the groups at any time (p>0.05, Figure 5). 

 Based on the count of collagen-positive points, the histological analysis of specimens demonstrated that Group 3 had the highest mean collagen content at all time points (Table 2). Group 2 also showed higher collagen content than Group 1 at all time points (Table 2). However, these differences were not statistically significant, as shown in Figure 5 and Table 2 (p>0.05). 

**Table 2:** Collagen density: descriptive analysis, separated by group and period. No statistically significant difference was observed among the groups (p>0.05).

		Mean	Median	SD	Minimum	Maximum
Day 7	Group 1	74.5	74.5	6.36	70	79
	Group 2	131	131	19	112	150
	Group 3	148.2	155	43.94	75	192
Day 14	Group 1	74.5	74.5	6.36	70	79
	Group 2	100	107	47.99	39	147
	Group 3	114	118.5	24.64	80	139
Day 56	Group 1	74.5	74.5	6.36	70	79
	Group 2	102	102	1.41	101	103
	Group 3	129.5	129.5	43.13	99	160

**Figure 6:**
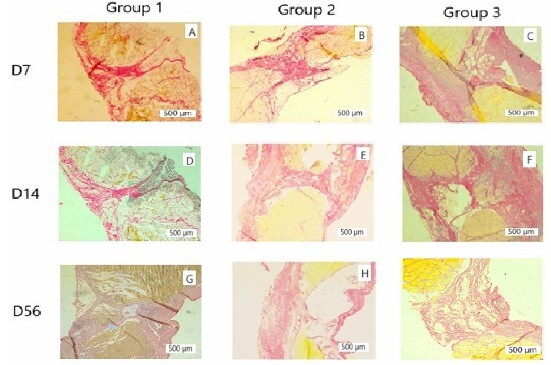
Representative photomicrographs of histological analyses of tissue samples stained with Picrosirius Red, organized by groups and analyzed periods. A, B, C: representative of D7, corresponding to groups 1, 2, and 3, respectively; D, E, F: representative of D14, corresponding to groups 1, 2, and 3, respectively; G, H, I: representative of D56, corresponding to groups 1, 2, and 3, respectively. Scale bar: 500 μm.

 Below are representative histological images of the midline of specimens from all groups at different time points (Figure 6). 

## DISCUSSION

 This study evaluated the potential effects of using mesenchymal stem cells derived from adipocytes to enhance tensile strength and collagen formation in rat aponeurosis. However, these parameters showed no improvement when stem cells were added to healthy rats. 

 Considering that adipose-derived mesenchymal stem cells have the ability to migrate to the injured site, we chose to use the suture coated with stem cells as their carrier because we had previously used this method, had experience with it, and had obtained satisfactory results. 

 The lack of difference observed between Groups 2 and 3 could raise questions as to whether the stem cells were indeed present on the suture and, if so, whether they were viable. To address this concern, we performed a Live and Dead assay, which confirmed the presence and viability of the stem cells on the suture. 

 One hypothesis for the lack of improvement is that wound healing is already optimized under normal conditions. Stem cells might be more beneficial in improving the healing of compromised tissues, such as those exposed to radiotherapy or in diabetic individuals^6^. 

 Most related research involves scaffolds or matrices populated with stem cells, but none have utilized suture filaments with adhered stem cells, as in the present study. 

 A study in Sprague-Dawley rats compared the use of sponges with and without bone marrow-derived stem cells. No differences in tensile strength between groups were found four weeks post-implantation^42^. Similarly, another study using collagen vehicles to deliver bone marrow-derived stem cells to rabbit aponeurosis did not report significant differences in tensile strength after eight weeks of implantation^26^. 

 The present results confirm previous findings by different authors. Mestak et al. used extracellular matrices with or without adipose tissue-derived mesenchymal stem cells in Wistar rats. After three months, their findings were mixed — some showed improved tensile strength, while others did not. The authors concluded that the use of stem cells was not advantageous^29^. 

 Conversely, Heffner et al. reported a statistically significant improvement in tensile strength after four and eight weeks when combining mesenchymal stem cells, platelet-rich plasma, and bovine collagen matrices in rats. This suggests a synergistic effect of the multiple components^13^. 

 Hansen et al.^12^ evaluated different meshes for pelvic floor reconstruction and observed an effect of the mesh itself on tensile strength. However, after mesh resorption at 24 weeks, there was no difference in tensile strength between groups with and without mesenchymal stem cells. 

 A study by van Steenberghe et al.^39^ used adipose- and bone marrow-derived mesenchymal stem cells adhered to a collagen matrix made from human aponeurosis implanted into rat abdominal walls. After 30 days, no difference in tensile strength was observed between groups with or without stem cells. However, the adipose-derived stem cell group significantly increased tissue vascularization. 

 A comprehensive review of mesenchymal stem cell use, particularly adipose-derived stem cells, in healing tendon injuries across species (rabbits and horses) reported improved tissue organization but inconsistent results in acute injuries and when combined with platelet-rich plasma^40^. 

 Another review analyzed stem cells attached to various types of mesh. Studies using biological and synthetic meshes found no improvement in tensile strength with stem cells, although vascularization did improve. The authors hypothesized that the inherent benefits of meshes could provide sufficient results^27^. 

 Finally, the two preceding studies that utilized this suture filament with adhered mesenchymal stem cells demonstrated its healing potential. The first study evaluated the efficacy of this suture filament in treating enterocutaneous fistulas. In contrast, the second combined the suture filament with a matrix containing mesenchymal stem cells for tracheal fistula repair. Both studies showed that this stem cell-enriched filament provided a statistically significant benefit in the healing process^25,41^. However, the main difference between these earlier and the present study lies in the tissues evaluated. The earlier studies primarily focused on epithelial tissues, such as the epidermis and respiratory epithelium, whereas this study evaluated connective tissue, specifically the aponeurosis. 

 Mestak et al. supported the hypothesis that stem cells exert their effects primarily on epithelial tissues. They demonstrated that rats treated with adipose-derived mesenchymal stem cells for abdominal wall hernia repair showed improved healing in the peritoneum but not in the aponeurosis^29^. Another study reported enhanced peritoneal formation with mesenchymal stem cell application^45^. Furthermore, Liu et al. found that adipose-derived mesenchymal stem cells improved epithelialization rates and collagen formation in cutaneous wounds in rats^24^. 

 Our study’s limitations include its exclusive focus on aponeurosis, without evaluating the peritoneum. Additionally, the histological analysis was limited to quantitative collagen assessment, without a qualitative evaluation of collagen types or other markers such as vascular endothelial growth factor (VEGF) and metalloproteinases, which are known to be influenced by stem cells. 

 Future studies could investigate the types of collagen, distinguishing between types I and III, as well as analyzing inflammatory markers and angiogenesis markers such as VEGF. Another possibility is the use of this mesenchymal stem cellenriched filament in immunocompromised animals, such as those subjected to chemotherapy, radiotherapy, or immunosuppressive medications. Moreover, this filament could be combined with matrices for reconstructive purposes in areas requiring extensive repair, such as large hernias where the aponeurosis is insufficient for defect correction. 

## CONCLUSIONS

 The present study’s data do not show that the use of mesenchymal stem cells improves the healing of a midline abdominal incision in otherwise healthy subjects. However, an option for future studies is to employ this filament, combined with matrices for reconstructive purposes, in areas requiring extensive repair, such as large hernias where the aponeurosis is insufficient for defect correction. 

## Data Availability

The Informations regarding the investigation, methodology and data analysis of the article are archived under the responsibility of the authors.
